# Weight change and risk of cardiovascular disease among adults with type 2 diabetes: more than 14 years of follow-up in the Tehran Lipid and Glucose Study

**DOI:** 10.1186/s12933-021-01326-2

**Published:** 2021-07-12

**Authors:** Seyyed Saeed Moazzeni, Reyhane Hizomi Arani, Niloofar Deravi, Mitra Hasheminia, Davood Khalili, Fereidoun Azizi, Farzad Hadaegh

**Affiliations:** 1 Prevention of Metabolic Disorders Research Center, Research Institute for Endocrine Sciences, Shahid Beheshti University of Medical Sciences, No. 24, Parvaneh Street, Velenjak, P.O. Box: 19395-4763, Tehran, Iran; 2 Student Research Committee, School of Medicine, Shahid Beheshti University of Medical Science, Tehran, Iran; 3 Department of Epidemiology and Biostatistics, Research Institute for Endocrine Sciences, Shahid Beheshti University of Medical Sciences, Tehran, Iran; 4 Endocrine Research Center, Research Institute for Endocrine Sciences, Shahid Beheshti University of Medical Sciences, Tehran, Iran

**Keywords:** Diabetes mellitus, type 2, Cardiovascular diseases, Coronary disease, Body weight changes, Sulfonylurea compounds

## Abstract

**Background:**

To examine the impact of weight change on incident cardiovascular disease and coronary heart disease (CVD/CHD) among an Iranian population with type 2 diabetes mellitus (T2DM).

**Methods:**

The study population included 763 participants with T2DM aged ≥ 30 years without a history of CVD and cancer at baseline. Two weight measurements done at baseline and about 3 years later. Based on their weight change, they categorized into: > 5% loss, 3–5% loss, stable (± < 3%), 3–5% gain, > 5% gain. Participants were then followed for incident CVD/CHD annually up to 20 March 2018. Multivariable Cox proportional hazard models, adjusted for age, sex, body mass index, educational level, current smoking, glucose-lowering drug use, family history of CVD, hypertension, hypercholesterolemia, chronic kidney disease, and fasting plasma glucose (FPG) were applied to estimate the hazard ratios (HRs) and 95% confidence intervals (CIs) of weight change categories for incident CVD/CHD, considering stable weight as reference.

**Results:**

After the weight change measurement, during a median follow-up of 14.4 years, 258 CVD and 214 CHD occurred. Over 5% weight gain was associated with reduced risks of CVD and CHD development by the HRs of 0.70 [95% CI 0.48–1.01; P-value: 0.058] and 0.61 [0.40–0.93], respectively, in multivariable analysis. After further adjustment for FPG change, the HRs of weight gain > 5% were attenuated to 0.75 [0.51–1.10; P-value: 0.138] and 0.66 [043–1.01; P-value: 0.053] for incident CVD and CHD, respectively. The effect of weight loss > 5% was in opposite direction among those older versus younger than 60 years; with suggestive increased risk (not statistically significant) of incident CHD/CVD for the older group. Moreover, weight gain > 5% significantly reduced the risk of CHD only among those older than 60 years (P-value for interaction < 0.2). Furthermore, weight gain > 5% had an association with lower risk of CVD and CHD among sulfonylurea users (0.56 [0.32–0.98] for CVD and 0.54 [0.29–0.99] for CHD).

**Conclusions:**

Our results with a long-term follow-up showed that weight gain > 5% was associated with better CVD/CHD outcomes among Iranian participants with T2DM, especially older ones. Moreover, we did not find an unfavorable impact on incident CVD/CHD for sulfonylurea-induced weight gain.

**Supplementary Information:**

The online version contains supplementary material available at 10.1186/s12933-021-01326-2.

## Background

The Middle East and North Africa (MENA) region had the second-highest prevalence of type 2 diabetes mellitus (T2DM) in 2017 globally (i.e., 10.8%), with an increasing pattern during the past three decades [1]. In 2011, nearly 4.5 million adults were living with diabetes in Iran, and it is estimated that 9.2 million Iranian adults will be affected by diabetes by the year 2030 [2]. This unceasing significant growth reveals a high burden of T2DM in Iran, particularly when considering its complications [2–4]. T2DM is the leading cause of cardiovascular disease (CVD) and mortality events among Iranian populations. Previously, we found that the population attributable fraction of T2DM for incident CVD events was 14% among Tehranian adults [5].

To improve hyperglycemia and cardiometabolic risk factors, intentional weight loss was recommended to overweight and obese patients with T2DM; however, the effect of weight loss on incident CVD events is less clear [6–8]. Moreover, unintentional weight loss is a marker of major health problems and sarcopenia; it can also be associated with increased morbidity and mortality, especially among older individuals [9]. Appropriate interventions to maintain a stable weight were also recommended to positively influence health outcomes, especially CVD outcomes, among patients with T2DM [10, 11]. Glucose-lowering drugs (GLDs) also can have effects on weight by inducing weight gain or weight loss among patients with T2DM [12].

There are few investigations about the impact of weight change on the risk of CVD events among populations with T2DM, with heterogenic designs and findings. A meta-analysis in 2018 reported that weight loss, but not weight gain, increased CVD mortality among individuals with T2DM; however, there was significant heterogeneity between the included studies (I^2^ = 98%) [13]. Results of the recent studies on this issue also remained inconsistent. Data from the ORIGIN trial and the Anglo–Danish–Dutch Study of Intensive Treatment in People with Screen-Detected Diabetes in Primary Care (ADDITION)—Cambridge trial showed significant protection for weight loss among participants with T2DM [14, 15]; however, the Action in Diabetes and Vascular disease: preterAx and diamicroN-MR Controlled Evaluation (ADVANCE) trial showed a significantly increased risk of CVD among participants with weight loss [16]. Moreover, results from other studies were not statistically significant for the association of weight loss with CVD [17–19]. Similar to weight loss, findings for the effect of weight gain on CVD events were inconclusive among populations with T2DM, in which adverse [16, 20], protective [15], and neutral [18, 19] effects have been reported for weight gain all together.

It also should be noted that previous studies on this issue were mainly limited to the US, European, and East Asian populations. Consequently, in the present study, we examined the impact of 3-year weight change on incident CVD/coronary heart disease (CHD) events among an Iranian population with T2DM during more than a decade of follow-up. Data from the oldest cohort of the MENA region, the Tehran Lipid and Glucose Study (TLGS), was used in this observational study.

## Methods

### Study design and study population

The TLGS is a prospective cohort study conducted on a general population in district 13 of Tehran. The first and second registration phases were on January 31, 1999–July 03, 2001 and October 20, 2001–September 22, 2005, respectively, and the cohort examinations were repeated every 3 years. The TLGS had an original aim of assessment the prevalence and incidence of non-communicable diseases and their risk factors. Also, the impact of changes in lifestyle factors was determined in this cohort. The design, measurement methods, and enrollment strategy of the study have been explained in detail by Azizi et al. [21]

From a total of 9558 participants aged ≥ 30 years, 1525 participants were individuals with T2DM. Firstly, we excluded 249 and 11 individuals with prevalent CVD and cancer at baseline, respectively, leading to 1265 participants. Those with missing data on weight measurement at baseline and first follow-up visit and missing data on relevant covariates were also excluded (n = 459). No follow-up information was another reason for exclusion (n = 43). Finally, we included 763 eligible subjects with T2DM (635 subjects from phase I and 128 new subjects from phase II) in our analysis.

**Fig. 1:**
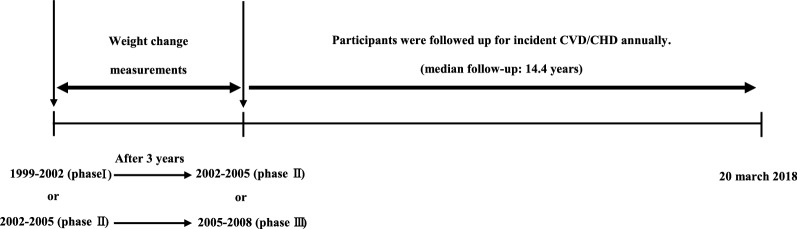
Timeline of the study design: Tehran Lipid and Glucose Study, Iran, 1999–2018

As shown in Fig. 1, for participants who were enrolled at phase I (baseline measurement), after about 3 years, phase II was set for follow-up measurement of weight. Moreover, for those enrolled at phase II (baseline measurement), follow-up measurement of weight was done at phase III (2005–2008). After these two weight measurements for weight change calculation, participants were followed-up annually for incident CVD/CHD up to 20 March 2018.

### Clinical and laboratory measurements

Demographic data, medication use, past medical history, family history of premature CVD, smoking habits, and educational level were acquired by validated and interviewer-administered questionnaires at visits. Weight was recorded by a digital scale to the nearest 100 g while participants had minimal clothes without shoes. Also, we assessed height without shoes in a standing position. Body mass index (BMI) was considered as weight in kilograms divided by height in meters squared. After 15 min of rest, systolic blood pressure (SBP) and diastolic blood pressure (DBP) were considered the mean of the two physician-measured blood pressure on the right arm using a standard sphygmomanometer. Morning blood samples were collected from all participants after at least 12 h of fasting. Participants without a history of using GLDs took orally 82.5 g glucose monohydrate solution (equivalent to 75 g anhydrous glucose), and their blood sample was taken after 2 h to assess 2-h post-challenge plasma glucose (2h-PCPG). The measurements of fasting plasma glucose (FPG), 2h-PCPG, total cholesterol, and serum creatinine were performed by standard methods, as previously explained [21].

### Definition of terms

In our study, T2DM was defined as one of these criteria: (a) FPG ≥ 7 mmol/L; (b) 2h-PCPG ≥ 11.1 mmol/L; (c) taking any GLDs; (d) self-reports of physician-diagnosed diabetes not on GLDs. Moreover, SBP ≥ 140 mmHg or DBP ≥ 90 mmHg or use of antihypertensive drugs was defined as hypertension. Also, using lipid-lowering drugs or having total cholesterol of ≥ 5.18 mmol/L was described as hypercholesterolemia. Glomerular filtration rate (GFR) was estimated by the Modification of Diet in Renal Disease (MDRD) study equation [22]. Chronic kidney disease (CKD) was defined as estimated GFR lower than 60 mL/min/1.73 m^2^. Educational levels were categorized into three groups: less than 6 years, between 6 and 12 years, and more than 12 years of formal education. We divided our participants based on the smoking status into two groups of current smokers and former/never smokers. We considered the positive family history of premature CVD in participants who have any history of stroke or CHD in female first-degree relatives < 65 years or male first-degree relatives < 55 years.

Weight change was calculated as: $\frac{{{\text{Follow-up}}\;{\text{measurement}} - {\text{Baseline}}\;{\text{measurement}}}}{{{\text{Baseline}}\;{\text{measurement}}}} \times 100$. Then participants were categorized into five groups based on 3-year weight change percentage: (i) more than 5% weight loss; (ii) 3% to 5% weight loss, (iii) less than 3% weight change (reference group: stable); (iv) + 3% to + 5% weight gain; (v) more than 5% weight gain, as recommended by Stevens et al. [23].

### Outcome assessment

Details of CVD/CHD data collection have been explained elsewhere [21]. Briefly, a trained nurse interviewed all participants through an annual phone call for any cardiovascular events. Furthermore, any reported event followed by a trained physician through home visits for data collection from medical documents or death certifications (in the cases of mortality). Finally, the outcome committee of the TLGS, consist of needed professionals such as an internist, an endocrinologist, a cardiologist, and an epidemiologist, evaluated the outcome data and adjudicated events. CHD included cases of certain myocardial infarction (MI) [diagnosed by electrocardiogram (ECG) and biomarkers including CK, CK-MB, CK-MBm, troponin (cTn), and myoglobin], probable MI [distinguished by positive ECG findings and cardiac symptoms or positive ECG findings with equivocal biomarkers], unstable angina pectoris [developed new cardiac symptoms or showed changing symptom patterns and positive ECG findings with normal biomarkers] angiographic-proven CHD [defined as ≥ 50% stenosis in at least one major coronary vessel], and cardiac death [any hospital death related to CHD based on the foregoing criteria or sudden cardiac death due to cardiac disease events less than 1 h after the beginning of symptoms based on verbal autopsy files outside of the hospital]. CVD was considered a composite measure of any CHD, fatal or non-fatal stroke [defined as a rapidly developing new focal or global neurological deficit lasting ≥ 24 h], and death due to cerebrovascular origin.

### Statistical analyses

Comparing baseline characteristics among respondents (study participants) versus non-respondents (including those with missing data of covariates or those without any follow-up data) was performed using Student’s *t*-test for continuous variables and chi-square test for categorical variables.

Baseline characteristics across weight change categories are illustrated as mean ± standard deviation (SD) for continuous variables and number (%) for categorical variables.

The multivariable Cox proportional regression analysis was applied to evaluate the association of weight change categories with incident CVD/CHD by reporting Hazard ratios (HRs) with 95% confidence intervals (CIs) in two models: Model 1: adjusted for age and sex; Model 2: further adjusted for BMI, educational level, current smoking (at first follow-up), GLDs use (at baseline or first follow-up), family history of premature CVD, hypertension, hypercholesterolemia, CKD, and FPG.

To address the low power and possibility of bias caused by missing data, as a sensitivity analysis, we examined the impact of weight change on incident CVD/CHD with imputed baseline and first follow-up missing data for covariates using stochastic single imputation with predictive mean matching (PMM) [24, 25].

Interactions of weight change categories with age groups (≥ 60 years versus < 60 years), sex (men versus women), BMI groups (≥ 30 kg/m^2^ versus < 30 kg/m^2^), and GLDs use (yes versus no) were checked by the log–likelihood ratio test in Model 2 and HRs with 95% CIs calculated for each subgroup.

Time to event was considered as the time of censoring or the outcome (incident CVD/CHD) occurring, whichever came first. We censored subjects in the case of leaving the district, lost to follow-up, or being without any event in the study until 20 March 2018.

Using the Schoenfeld residual test, the proportionality in the Cox model was evaluated; all proportionality assumptions were appropriate. STATA version 14 (StataCorp LP, College Station, Texas) was employed for statistical analyses. A P-value of < 0.05 and P-value for interaction of < 0.2 was considered to be statistically significant.

## Results

**Fig. 2:**
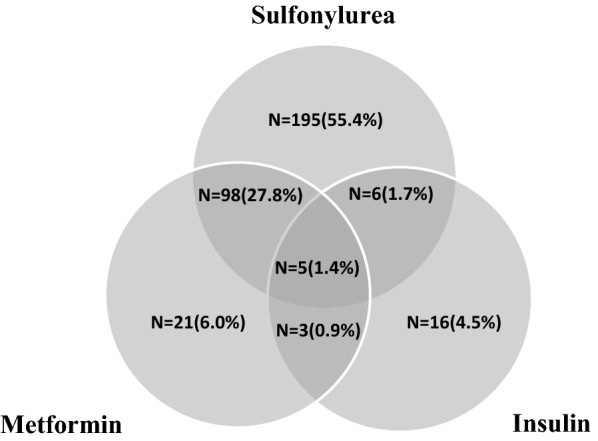
Number of patients with different types of GLDs at baseline or first follow-up. Percentage of each group was calculated only among 352 patients who had been on GLDs at baseline or first follow-up, considering that for 8 patients, type of GLDs that they had used was missed. *N* number, *GLD* glucose lowering drug

As presented in Additional file 1: Table S1, there was no significant difference between respondents and non-respondents except that respondents were about 2 years younger. The study population included 763 individuals (300 men) with T2DM. The mean age was 53.6 (SD: 11.0) years at baseline. Moreover, 352 subjects (46% of the total population) had been on GLDs at baseline or first follow-up. As shown in Fig. 2, 304 participants (86.4% of total GLD users) had used sulfonylureas only or in combination with metformin and/or insulin. The baseline and the first follow-up characteristics of the participants across weight change categories are shown in Table 1. About 45% of our participants had a stable weight (− 3% to + 3%) during the first three years of follow-up. Moreover, 16.1% and 17.3% of the total participants had more than 5% weight loss and weight gain, respectively. Weight gain > 5% was associated with reduced FPG level after 3 years, but having a weight loss > 3% was accompanied by elevated FPG after 3 years.

**Table 1:** Baseline and first follow-up characteristics of participants with T2DM across weight change categories at the baseline and after 3-year follow-up: Tehran Lipid and Glucose Study, Iran, 1999–2018

Weight change categories	Lost > 5%		Lost 3% to 5%		Stable (± 3%)		Gained 3% to 5%		Gained > 5%		Total	
Number of participants (men)	123 (46)		87 (38)		348 (146)		73 (30)		132 (40)		763 (300)	
	Baseline	Follow-up	Baseline	Follow-up	Baseline	Follow-up	Baseline	Follow-up	Baseline	Follow-up	Baseline	Follow- up
Continuous variables, mean ± SD												
Age (year)	55.1 ± 11.0	58.4 ± 11.1	53.5 ± 10.8	56.8 ± 11.1	53.6 ± 10.9	56.8 ± 10.9	51.9 ± 10.7	54.9 ± 10.7	53.1 ± 11.2	56.6 ± 11.2	53.6 ± 11.0	56.8 ± 11.0
BMI (kg/m^2^)	29.9 ± 4.5	27.7 ± 4.4	29.7 ± 5.0	28.7 ± 4.8	29.0 ± 4.0	29.1 ± 4.1	28.0 ± 4.2	29.0 ± 4.5	27.9 ± 5.0	30.7 ± 5.4	28.9 ± 4.4	29.1 ± 4.6
SBP (mmHg)	134.4 ± 21.6	129.8 ± 20.2	135.5 ± 23.9	133.7 ± 22.7	132.7 ± 22.0	130.8 ± 21.0	130.7 ± 22.6	129.9 ± 22.3	128.9 ± 22.1	131.3 ± 23.3	132.4 ± 22.3	131.0 ± 21.6
DBP (mmHg)	84.0 ± 12.1	78.4 ± 11.7	83.0 ± 11.6	78.9 ± 11.5	82.3 ± 11.6	79.4 ± 10.5	81.6 ± 10.1	78.9 ± 9.2	80.6 ± 11.5	78.3 ± 11.7	82.3 ± 11.5	79.0 ± 10.9
FPG (mmol/L)^a^	9.1 ± 3.1	9.8 ± 4.2	9.1 ± 3.4	9.4 ± 3.8	8.6 ± 3.2	8.6 ± 3.2	7.5 ± 3.2	7.5 ± 2.5	8.2 ± 3.9	7.8 ± 2.9	8.6 ± 3.4	8.7 ± 3.4
Total cholesterol (mmol/L)^a^	6.2 ± 1.4	5.5 ± 1.2	5.8 ± 1.1	5.4 ± 1.0	5.9 ± 1.3	5.4 ± 1.2	5.8 ± 1.0	5.5 ± 0.9	5.8 ± 1.3	5.4 ± 1.2	5.9 ± 1.3	5.4 ± 1.1
e-GFR (mL/min/1.73 m^2^)	63.2 ± 10.4	66.4 ± 9.5	67.6 ± 11.6	69.3 ± 12.1	65.6 ± 10.7	69.4 ± 13.0	65.2 ± 11.1	71.4 ± 12.6	66.9 ± 12.5	68.2 ± 13.2	65.6 ± 11.2	68.9 ± 12.5
Categorical variables, number (%)												
Educational level, years												
< 6	83 (67.5)	78 (63.4)	44 (50.6)	47 (54.7)	219 (62.9)	217 (62.5)	41 (56.2)	42 (57.5)	78 (59.1)	79 (59.8)	465 (60.9)	463 (60.8)
6–12	29 (23.6)	35 (28.5)	35 (40.2)	30 (34.9)	106 (30.5)	106 (30.5)	27 (37.0)	26 (35.6)	49 (37.1)	48 (36.4)	246 (32.2)	245 (32.2)
> 12	11 (8.9)	10 (8.1)	8 (9.2)	9 (10.5)	23 (6.6)	24 (6.9)	5 (6.8)	5 (6.8)	5 (3.8)	5 (3.8)	52 (6.8)	53 (7.0)
Current smoker	7 (5.7)	6 (4.9)	11 (12.6)	11 (12.6)	34 (9.8)	33 (9.5)	10 (14.1)	9 (12.3)	13 (9.9)	10 (7.6)	75 (9.9)	69 (9.0)
Family history of premature CVD, yes	28 (22.8)	32 (26.0)	17 (19.5)	22 (25.3)	64 (18.4)	79 (22.7)	16 (21.9)	23 (31.5)	19 (14.4)	25 (18.9)	144 (18.9)	181 (23.7)
GLD use, yes	47 (38.2)	51 (41.5)	27 (31.0)	40 (46.0)	104 (29.9)	149 (42.8)	22 (30.1)	26 (35.6)	36 (27.3)	59 (44.7)	236 (30.9)	325 (42.6)
Anti-hypertensive drug use, yes	29 (23.6)	31 (25.2)	22 (25.3)	23 (26.4)	56 (16.1)	67 (19.3)	9 (12.3)	11 (15.1)	27 (20.5)	30 (22.7)	143 (18.7)	162 (21.2)
Lipid-lowering drug use, yes	14 (11.4)	13 (10.6)	11 (12.6)	16 (18.4)	36 (10.3)	40 (11.5)	5 (6.8)	4 (5.5)	7 (5.3)	13 (9.8)	73 (9.6)	86 (11.3)

For participants that enrolled at phase I (1999–2001) of TLGS, phase II (2001–2005) was considered as the follow-up for weight change calculation. For participants that enrolled at phase II (2001–2005) of TLGS, phase III (2005–2008) was considered as the follow-up for weight change calculation

*T2DM* type 2 diabetes mellitus, *SD* standard deviation, *BMI* body mass index, *SBP* systolic blood pressure, *DBP* diastolic blood pressure, *FPG* fasting plasma glucose, *e-GFR* estimated glomerular filtration rate, *CVD* cardiovascular disease, *GLD* glucose-lowering drug

^a^Conversion factors from mmol/L to mg/dL were 18.02 for FPG and 38.67 for total cholesterol

During a median follow-up of 14.4 years after the weight change measurement [interquartile range: 12.1–15.5], 258 CVD (CHD:214) occurred. Multivariable HRs and 95% CIs of association between weight change categories and incident CVD/CHD events are presented in Tables 2 and 3. Compared to those with stable weight, gaining weight more than 5% had sex- and age-adjusted HRs of 0.71 [95% CI 0.49–1.03; P-value: 0.075] for incident CVD and 0.62 [0.41–0.94] for incident CHD. After controlling for the potential confounding factors in model 2, these risks became 0.70 [0.48–1.01; P-value: 0.058] for CVD and 0.61 [0.40–0.93] for CHD. Moreover, after adjustment for FPG change during the first 3 years of follow-up, the effect size of weight gain > 5% was attenuated to 0.75 [0.51–1.10; P-value: 0.138] and 0.66 [043–1.01; P-value: 0.053] for incident CVD and CHD, respectively. Generally, older age, current smoking, GLD use, hypertension, hypercholesterolemia, and FPG were associated with higher risk of incident CVD/CHD in the model 2.

**Table 2:** Multivariable hazard ratios (HR) and 95% confidence intervals (CI) of association between weight change categories and incident CVD: Tehran Lipid and Glucose Study, Iran, 1999–2018

	Model 1		Model 2	
	HR (95% CI)	P-value	HR (95% CI)	P-value
Weight change categories				
Lost > 5%	1.18 (0.84–1.65)	0.333	1.11 (0.79–1.56)	0.538
Lost 3% to 5%	1.04 (0.70–1.53)	0.863	0.89 (0.60–1.33)	0.571
Stable (± 3%)	Reference		Reference	
Gained 3% to 5%	0.69 (0.42–1.11)	0.128	0.76 (0.46–1.23)	0.260
Gained > 5%	0.71 (0.49–1.03)	0.075	0.70 (0.48–1.01)	0.058
Age, year	1.05 (1.04–1.06)	< 0.001	1.05 (1.03–1.07)	< 0.001
Women (men as reference)	0.90 (0.70–1.15)	0.394	0.78 (0.58–1.05)	0.101
BMI, kg/m^2^			1.00 (0.97–1.03)	0.894
Educational level, years				
> 12			Reference	
6–12			0.81 (0.47–1.38)	0.437
< 6			0.82 (0.48–1.39)	0.459
Current smoker, yes			1.64 (1.03–2.61)	0.036
GLD use, yes			1.62 (1.21–2.16)	0.001
Family history of premature CVD, yes			1.15 (0.84–1.57)	0.389
Hypertension, yes			1.73 (1.32–2.26)	< 0.001
Hypercholesterolemia, yes			1.77 (1.27–2.48)	0.001
CKD, yes			0.78 (0.58–1.05)	0.097
FPG, mmol/L			1.04 (1.00–1.08)	0.039

Model 1: adjusted for age and sex. Model 2: Model 1 + further adjusted for BMI, educational level, current smoking (at first follow-up), GLD use (at baseline or first follow-up), family history of premature CVD, hypertension, hypercholesterolemia, CKD, and FPG

*CVD* cardiovascular disease, *BMI* body mass index, *GLD* glucose-lowering drugs, *CKD* chronic kidney disease, *FPG* fasting plasma glucose

**Table 3:** Multivariable hazard ratios (HR) and 95% confidence intervals (CI) of association between weight change categories and incident CHD: Tehran Lipid and Glucose Study, Iran, 1999–2018

	Model 1		Model 2	
	HR (95% CI)	P-value	HR (95% CI)	P-value
Weight change categories				
Lost > 5%	1.19 (0.83–1.71)	0.334	1.14 (0.79–1.65)	0.471
Lost 3% to 5%	0.86 (0.55–1.36)	0.526	0.76 (0.48–1.20)	0.238
Stable (± 3%)	Reference		Reference	
Gained 3% to 5%	0.63 (0.37–1.08)	0.096	0.66 (0.38–1.15)	0.141
Gained > 5%	0.62 (0.41–0.94)	0.026	0.61 (0.40–0.93)	0.021
Age, year	1.04 (1.03–1.05)	< 0.001	1.04 (1.02–1.05)	< 0.001
Women (men as reference)	0.92 (0.70–1.22)	0.569	0.82 (0.59–1.13)	0.216
BMI, kg/m^2^			0.99 (0.96–1.02)	0.423
Educational level, years				
> 12			Reference	
6–12			0.91 (0.50–1.66)	0.768
< 6			0.92 (0.51–1.69)	0.796
Current smoker, yes			1.73 (1.06–2.83)	0.028
GLD use, yes			1.75 (1.27–2.41)	0.001
Family history of premature CVD, yes			1.08 (0.76–1.52)	0.672
Hypertension, yes			1.63 (1.22–2.19)	0.001
Hypercholesterolemia, yes			2.09 (1.43–3.05)	< 0.001
CKD, yes			0.76 (0.55–1.05)	0.099
FPG, mmol/L			1.03 (0.99–1.08)	0.139

Model 1: adjusted for age and sex. Model 2: Model 1 + further adjusted for BMI, educational level, current smoking (at first follow-up), GLD use (at baseline or first follow-up), family history of premature CVD, hypertension, hypercholesterolemia, CKD, and FPG

*CHD* coronary heart disease, *BMI* body mass index, *GLD* glucose-lowering drugs, *CVD* cardiovascular disease, *CKD* chronic kidney disease, *FPG* fasting plasma glucose

**Fig. 3:**
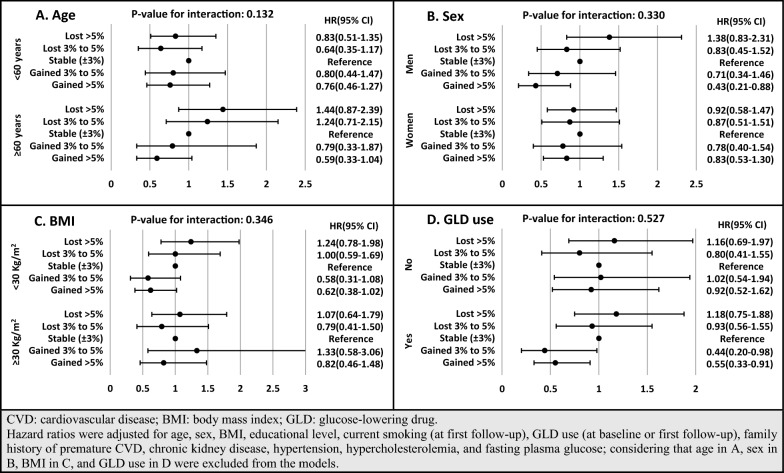
Multivariable hazard ratios (HR) and 95% confidence intervals (CI) of association between weight change categories and incident CVD, stratified by age (**A**), sex (**B**), BMI (**C**), and GLD use (**D**): Tehran Lipid and Glucose Study, Iran, 1999–2018

**Fig. 4:**
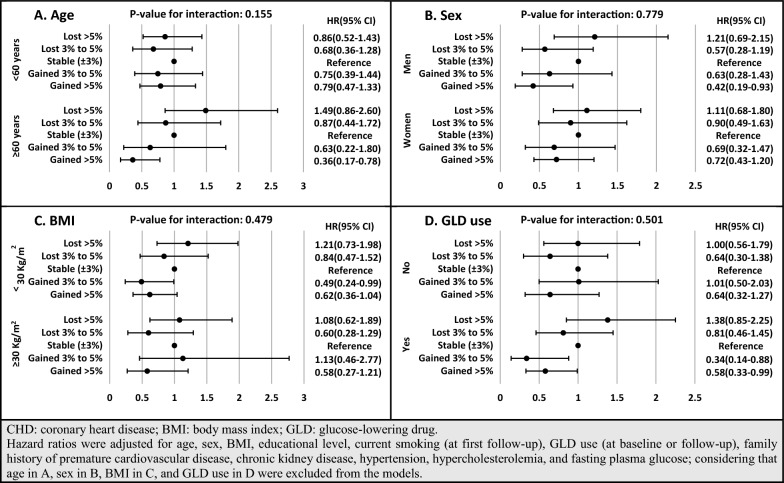
Multivariable hazard ratios (HR) and 95% confidence intervals (CI) of association between weight change categories and incident CHD, stratified by age (**A**), sex (**B**), BMI (**C**), and GLD use (**D**): Tehran Lipid and Glucose Study, Iran, 1999–2018

**Fig. 5:**
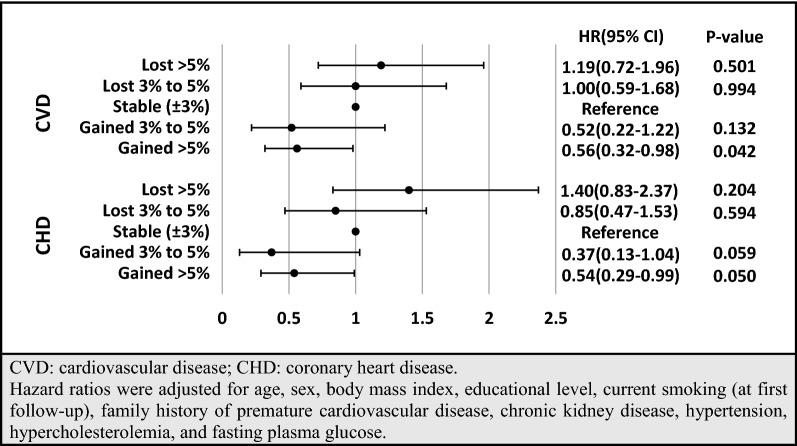
Multivariable hazard ratios (HR) and 95% confidence intervals (CI) of association between weight change categories and incident CVD/CHD among sulfonylurea users: Tehran Lipid and Glucose Study, Iran, 1999–2018

To show the robustness of our findings, a series of sensitivity analyses was performed. First, as presented in Additional file 2: Table S2 and Additional file 3: Table S3, based on our results from imputed baseline missing data for covariates (participant number: 1104) with the occurrence of 341 cases of incident CVD (286 CHD cases), our findings were consistent with the main analysis. Accordingly, more than 5% weight gain decreased the risk of incident CVD and CHD in model 2 with HRs of 0.69 [0.49–0.96] and 0.62 [0.43–0.90], respectively. After adjustment for FPG change, the HRs of weight gain > 5% were attenuated to 0.76 [0.54–1.05; P-value: 0.100] and 0.67 [0.46–0.98]. Second, multivariable HRs and 95% CIs for different weight change categories, stratified by age, sex, BMI, and GLD use, are shown in Figs. 3 and 4. Considering age stratification, for those aged < 60 years, no significant difference was found between weight change categories; however, among participants aged ≥ 60 years, more than 5% weight gain had HRs of 0.59 [0.33–1.04; P-value: 0.069] and 0.36 [0.17–0.78] for incident CVD and CHD, respectively. Moreover, weight loss > 5% showed higher risk among older and lower risk among younger participants; however, it did not reach a significant level (P-value for the interaction of age < 0.2). Moreover, although the interactions of weight change categories with sex, BMI groups, and GLD use were not significant, weight gain > 5% among men and weight gain > 3% among GLD users also had significantly lower risk of CVD/CHD. Third, since sulfonylurea was the most commonly used GLD in our population, we examined the association between weight change categories with incident CVD/CHD among users of sulfonylureas (Fig. 5). Those with weight gain of over 5% were at lower risk of incident CVD and CHD. Moreover, participants with weight gain of 3–5% had a marginally significant HR of 0.37 [0.13–1.04; P-value: 0.059] for incident CHD.

## Discussion

In this cohort study, during a long-term follow-up, after adjustment for well-known CVD risk factors, more than 5% weight gain within 3 years was associated with reduced risk of CVD/CHD development among an Iranian population with T2DM. However, after adjustment for FPG change, the effect sizes of the associations were attenuated to some degree but remained significant for CHD. We also found significant effect modification of age in the association between weight change and incident CVD/CHD; weight gain > 5% reduced the risk of incident CHD significantly only among those older than 60 years. Although there is no significant interaction between weight change categories and GLD use, over 3% weight gain reduced the risk of incident CVD/CHD among GLD users.

It should be noted that previous studies with similar aims to ours calculated weight or BMI change in different time intervals and categorizations. These studies also varied in study design, study setting (clinical-based [15, 19] versus population-based [17, 26]), and other aspects of methodology. Therefore, it is difficult to compare our findings with other studies in this field.

In contrast to our results, a flat V-shaped association was observed between BMI change and cardiac death or non-fatal MI in the Action to Control Cardiovascular Risk in Diabetes (ACCORD) study, in which stable weight had the lowest risk; the risk increased slowly as the weight was either increased or decreased; however, there is no significant difference between BMI change categories and the risk of these outcomes [19]. Moreover, among Swedish subjects with T2DM, an increased BMI during 18 months led to higher risks of CVD mortality (HR: 1.63 [1.11–2.39]) [20].

Similar to our results, data from the ORIGIN trial showed that among participants with T2DM or prediabetes, 1-year weight gain and weight loss, respectively, were associated with decreased and increased risk of an outcome composite of the first occurrence of cardiovascular death, non-fatal myocardial infarction, or non-fatal stroke during over 6 years of follow-up [15]. In a meta-analysis of prospective studies, despite a high heterogeneity, weight loss increased the risk of CVD mortality by 15% among overweight or obese patients with diabetes; moreover, weight gain had a HR of 0.97 [0.93–1.01] [13]. Based on results from the ADVANCE trial, compared to a stable weight, 2-year weight loss > 10% was associated significantly with increased risk of major cardiovascular events and CVD mortality; in contrast, weight gain did not contain a significant effect on CVD [16]. Recently, among about 20,000 White British UK biobank participants with T2DM, it was shown that the minimum mortality risk was seen in those with a BMI of 32 kg/m^2^; moreover, using Mendelian randomization, the researchers did not find a significant impact of obesity on mortality in this population [27]. Generally, for weight gain, we did not find an unfavorable impact on CVD/CHD among subjects with T2DM in TLGS, and over 5% weight gain was significantly associated with lower risk of incident CVD and CHD. However, our results should not translate into a recommendation for weight gain among individuals with T2DM. Our study was an observational study, and there was no intervention for intentional weight change (gain or loss). Despite this fact, it seems that weight management among diabetic individuals remained challenging. Focusing on intentional weight change among overweight or obese patients with T2DM, based on data from the Look AHEAD (Action for Health in Diabetes) study, which compared the effect of intensive lifestyle intervention with a control group that received diabetes support and education, it was shown that 1-year weight loss was associated with significant improvements in CVD risk factors, with greater benefits in a larger range of weight loss and more prominent in the intervention group [28]; however, this intensive lifestyle intervention did not reduce the risk of incident CVD [7]. Moreover, results for the effect of bariatric surgery among obese individuals with T2DM have consistently shown improvement in cardiovascular risk factors; however, randomized controlled trials did not have adequate power to assess improvement in CVD outcomes [29].

In the current study, although the interaction between GLD usage and weight change categories was not significant, the effect of weight gain on CVD development was more prominent among GLDs users. In our data set, GLD users, mainly included users of sulfonylureas at the recruitment time, had a higher risk for incident CVD/CHD (Tables 2 and 3); however, considering the lack of data on the duration of diabetes for our participants, this finding cannot be conclusively translated into the adverse effect of sulfonylureas or other types of GLDs on incident CVD/CHD, because our GLD users were known-diabetic patients with potentially more prolonged duration of disease and worse baseline characteristics rather than not GLD users (allocation bias) [30]. Furthermore, due to weight gain and hypoglycemia induced by sulfonylureas, there is still a debate about their long-term cardiovascular safety [30–33]; however, it is still recommended to clinicians to continue prescription of low-cost sulfonylureas in T2DM, with confidence in their effectiveness for prevention of microvascular complications and their potential cardiovascular safety [30, 34]. Now our findings reduced this concern about sulfonylurea-induced weight gain by showing that weight gain > 5% not only did not increase the risk of incident CVD/CHD but also it could decrease the risk among our sulfonylurea users. Similar to our finding for sulfonylureas, other previous studies showed no adverse effect on CVD outcomes for insulin- and pioglitazone-induced weight gain [35, 36]. The main explanation for this risk-reducing effect of weight gain among GLDs users might be that weight gain can be an indicator of improved patients’ compliance, good response to management, and preserved beta-cell function [37]. In support of this idea, in our data analysis, after adjustment for FPG change, the HRs of the association between weight gain and risk of CVD/CHD were attenuated, especially for incident CVD. It means that FPG change played the role of mediator for this association. Similarly, among Iranian patients with T2DM that mostly had been on GLDs, Janghorbani et al. also found weight gain to be accompanied by decreased FPG and glycosylated hemoglobin (HbA1c), which reflect improved glycemic control [38]. Importantly, new GLDs, especially analog GLP-1 and SGLT2i, have evidence-based recommendations for the managements of T2DM; however, as emphasized in different guidelines (such as American Diabetes Association), in situations that “cost is a major issue”, metformin, and sulfonylureas still can be considered as a first-line therapy [34]. Moreover, in our country, the new medications are not covered by insurance companies, and only a limited number of Iranians with T2DM can use them now. Moreover, in our data set, we reassessed the situation of GLDs in 2015–2018 (phase VI of TLGS) and found that 41% of GLDs users still took sulfonylureas (data not shown).

On the other hand, among our new cases of T2DM (those diagnosed by screening at baseline and had not used GLDs), no weight change category was related to incident CVD/CHD. It should be noted that at the baseline requirement, more than half of our diabetic population were newly diagnosed cases that were screened by FPG or 2h-PCPG. Despite this, we acknowledged that diabetes awareness in our population was poor at enrollment, which is consistent with previous reports in Iran [39, 40]. Focusing on newly-diagnosed cases of T2DM, result from observational analysis of the ADDITION-Europe trial also showed that there is no evidence of a significant association between 1-year and 5-year weight change categories and incident CVD [18]; however, considering data from Cambridge center, it was shown that in addition to the significant protective effect of weight loss ≥ 5%, weight gain > 2% also had a suggestive (not statistically significant) protective association with incident CVD, during 10 years of follow-up [14]. Additionally, among Scottish and German newly-diagnosed T2DM cases, respectively, weight change and BMI change were not related to macrovascular outcomes [26, 41]. A study among Korean participants also reported that a 2-year weight gain of ≥ 10% after the diagnosis of T2DM could not affect the risk of MI; however, it was introduced as a risk factor for stroke (HR:1.47 [1.20, 1.79] in the full-adjusted model) [17].

In our data analysis, not only > 5% weight gain was strongly associated with reduced risk of incident CHD among adults older than 60 years, but also weight loss > 5% showed a suggestive increased risk of incident CVD/CHD among the older-aged group only. Similarly, the impact of obesity on CVD was also different between younger and older adults in some other studies [42, 43], in which the unfavorable impact of obesity attenuated in older groups. Moreover, in contrast to younger adults, it is not clear that obese older adults should be recommended for losing their weight (intentional weight loss) [6, 44]. Unintentional weight loss among older adults could also reflect an advanced or chronic illness, which caused a loss of muscle mass and strength (sarcopenia) [45], a condition that can be an independent risk factor for CVD development [46]. Conversely, weight gain may be a feature of neutralizing catabolic dominance and restored anabolic activity.

The current study has several strengths. First, most studies related to the effects of weight change were based on self-reported questionnaires, which may have a recall information bias; however, our study used actual measurements based on the physical examination during every follow-up. Second, most previous studies that have a similar object with us considered prevalent CVD at baseline as a covariate [15, 16, 19], but we excluded them, and our outcome was the first occurrence of CVD events. We also had several limitations in our study. First, intentional weight loss was suggested to be associated with a lower CVD incidence rate [47, 48]; however, as the TLGS protocol did not include any specific weight change intervention for weight modification, we did not know whether weight changes of our participants were intentional or unintentional. Second, measures of weight change do not differentiate between changes in lean or fat mass; decreased muscle mass (sarcopenia) has an association with higher risk for mortality and CVD [46, 49]. Third, we did not have data on the duration of diabetes; however, more than half of our diabetic population were newly diagnosed ones. Fourth, considering the cost and lack of precise method, the HbA1C was not measured for participants; however, we adjusted our models for FPG as surrogates of HbA1C level [50]. Fifth, different tools were used for physical activity level assessment in phases I (Lipid Research Clinic questionnaire) and II (Modifiable Activity Questionnaire) [21]. Therefore, we were not able to consider physical activity and its change as covariates. Sixth, variables were considered at the baseline phases (or first follow-up), and possible changes were not taken into account from baseline up to incident CVD/CHD. Seventh, because of limited outcome numbers, we were not able to examine the association of weight change categories with different subtypes of CVD, including MI, heart failure, and stroke, separately. Finally, our study population is limited to residents of Tehran, a metropolitan city, with uniform ethnicity; hence our findings may not be generalizable to rural populations or other ethnicities.

## Conclusion

Our results with a long term follow-up showed that compare to stable weight (± 3%), 3-year weight gain > 5% can be associated with better CVD/CHD outcomes among Iranian participants with T2DM, especially those who were older. Our findings shed light on the heterogeneous effect of weight change on CVD/CHD events among subjects with T2DM. Further researches, especially clinical trials, are needed to examine the effect of intentional and unintentional weight change on common adverse outcomes among diabetic populations. Moreover, despite an already existed concern about the effect of sulfonylureas on weight gain, we did not find an unfavorable impact on CVD events for sulfonylurea-induced weight gain.

## Supplementary Information

**Additional file 1: Table S1.** Baseline characteristics of the respondents (study participants) and non-respondents: Tehran Lipid and Glucose Study, Iran, 1999–2018.

**Additional file 2: Table S2.** Multivariable hazard ratios (HR) and 95% confidence intervals (CI) of association between weight change categories and incident CVD with imputed baseline missing data for covariates (number: 1104 participants): Tehran Lipid and Glucose Study, Iran, 1999–2018.

**Additional file 3: Table S3.** Multivariable hazard ratios (HR) and 95% confidence intervals (CI) of association between weight change categories and incident CHD with imputed baseline missing data for covariates (number: 1104 participants): Tehran Lipid and Glucose Study, Iran, 1999–2018.

## Data Availability

The datasets used and analyzed during the current study are available from the corresponding author on reasonable request.

## Abbreviations

MENA: Middle East and North Africa
T2DM: Type 2 diabetes mellitus
CVD: Cardiovascular disease
GLD: Glucose-lowering drug
ADDITION: Anglo–Danish–Dutch Study of Intensive Treatment in People with Screen-Detected Diabetes in Primary Care
ADVANCE: Action in Diabetes and Vascular disease: PreterAx and diamicroN-MR Controlled Evaluation
CHD: Coronary heart disease
TLGS: Tehran Lipid and Glucose Study
BMI: Body mass index
SBP: Systolic blood pressure
DBP: Diastolic blood pressure
2h-PCPG: 2-Hour post-challenge plasma glucose
FPG: Fasting plasma glucose
GFR: Glomerular filtration rate
MDRD: Modification of Diet in Renal Disease
CKD: Chronic kidney disease
MI: Myocardial infarction
ECG: Electrocardiogram
SD: Standard deviation
HR: Hazard ratios
CI: Confidence interval
PMM: Predictive mean matching
ACCORD: Action to Control Cardiovascular Risk in Diabetes
HbA1c: Glycosylated hemoglobin
