# Supplementary material for: Weight change and risk of cardiovascular disease among adults with type 2 diabetes: more than 14 years of follow-up in the Tehran Lipid and Glucose Study
Source: Cardiovasc Diabetol. 2021 Jul 12;20:141. doi: 10.1186/s12933-021-01326-2 (PMC8276460; doi:10.1186/s12933-021-01326-2)
Supplement: Supplementary file 1 — Additional file 1: Table S1. Baseline characteristics of the respondents (study participants) and non-respondents: Tehran Lipid and Glucose Study, Iran, 1999–2018. [file 12933_2021_1326_MOESM1_ESM.pdf]

Table S1. Baseline characteristics of the respondents (study participants) and non-respondents: Tehran Lipid and Glucose Study, Iran, 1999-2018.

	Respondents	Non-respondents	P-value
**Number of participants**	**763**	**502**	
**Continuous variables, Mean ± SD**			
Age (year)	53.6 ± 11.0	55.3 ± 11.9	0.008
BMI (kg/m^2)	28.9 ± 4.4	28.7 ± 4.9	0.406
SBP (mmHg)	132.4 ± 22.3	134.0 ± 23.2	0.230
DBP (mmHg)	82.3 ± 11.5	82.0 ±11.3	0.628
FPG (mmol/L)*	8.6 ± 3.4	8.8 ± 3.7	0.378
Total cholesterol (mmol/L)*	5.9 ± 1.3	6.0 ± 1.3	0.668
GFR (mL/min/1.73 m^2)	65.6 ± 11.2	65.1 ± 12.1	0.435
**Categorical variables, number (%)**			
Educational level, years			0.452
- <6	465(60.9)	298(59.5)	
- 6-12	246(32.2)	165(32.9)	
- >12	52(6.8)	38(7.6)	
Current smoker	75(9.9)	63(13.1)	0.078
Family history of premature CVD, yes	144(18.9)	99(19.7)	0.708
GLD use, yes	236(30.9)	170(33.9)	0.274
Anti-hypertensive drug use, yes	143(18.7)	15.9(80)	0.200
Lipid-lowering drug use, yes	73(9.6)	64(12.7)	0.075

BMI: body mass index; SBP: systolic blood pressure; DBP: diastolic blood pressure; FPG: fasting plasma glucose; GFR: glomerular filtration rate; CVD: cardiovascular disease; GLD: glucose lowering drug.

Values are shown as Mean ± SD and number (%) for continuous and categorical variables, respectively.

*Conversion factors from mmol/L to mg/dL were 18.02 for FPG and 38.67 for total cholesterol.
