# Supplementary material for: Weight change and risk of cardiovascular disease among adults with type 2 diabetes: more than 14 years of follow-up in the Tehran Lipid and Glucose Study
Source: Cardiovasc Diabetol. 2021 Jul 12;20:141. doi: 10.1186/s12933-021-01326-2 (PMC8276460; doi:10.1186/s12933-021-01326-2)
Supplement: Supplementary file 2 — Additional file 2: Table S2. Multivariable hazard ratios (HR) and 95% confidence intervals (CI) of association between weight change categories and incident CVD with imputed baseline missing data for covariates (number: 1104 participants): Tehran Lipid and Glucose Study, Iran, 1999–2018. [file 12933_2021_1326_MOESM2_ESM.docx]

| **Table S2. Multivariable hazard ratios (HR) and 95% confidence intervals (CI) of association between weight change categories and incident CVD with imputed baseline missing data for covariates (number: 1104 participants): Tehran Lipid and Glucose Study, Iran, 1999-2018.** | | | | |
| --- | --- | --- | --- | --- |
|  | **Model 1** | | **Model 2** | |
|  | **HR (95% CI)** | **P-value** | **HR (95% CI)** | **P-value** |
| **Weight change categories** |  |  |  |  |
| - **Lost >5%** | 1.10 (0.82-1.48) | 0.522 | 1.07 (0.79-1.44) | 0.677 |
| - **Lost 3% to 5%** | 0.97 (0.68-1.39) | 0.859 | 0.87 (0.61-1.25) | 0.454 |
| - **Stable (±3%)** | Reference |  | Reference |  |
| - **Gained 3% to 5%** | 0.73 (0.49-1.09) | 0.122 | 0.85 (0.57-1.26) | 0.422 |
| - **Gained >5%** | **0.69 (0.49-0.95)** | **0.024** | **0.69 (0.49-0.96)** | **0.029** |
| **Age, year** | **1.05 (1.04-1.06)** | **<0.001** | **1.04 (1.03-1.05)** | **<0.001** |
| **Women (Men as reference)** | **0.77 (0.62-0.96)** | **0.018** | **0.62 (0.48-0.79)** | **<0.001** |
| **BMI, kg/m^2^** |  |  | 1.00 (0.97-1.02) | 0.811 |
| **Educational level, years** |  |  |  |  |
| - **>12** |  |  | Reference |  |
| - **6-12** |  |  | 0.92 (0.57-1.47) | 0.716 |
| - **<6** |  |  | 0.98 (0.61-1.57) | 0.944 |
| **Current smoker, yes** |  |  | 1.39 (0.93- 2.08) | 0.105 |
| **GLD use, yes** |  |  | **1.69 (1.32-2.15)** | **<0.001** |
| **Family history of premature CVD, yes** |  |  | 1.11 (0.84-1.46) | 0.473 |
| **Hypertension, yes** |  |  | **1.86 (1.47-2.34)** | **<0.001** |
| **Hypercholesterolemia, yes** |  |  | **1.75 (1.31-2.33)** | **<0.001** |
| **CKD, yes** |  |  | 0.82 (0.63-1.05) | 0.118 |
| **FPG, mmol/L** |  |  | **1.05 (1.02-1.09)** | **0.002** |
| CVD: cardiovascular disease; BMI: body mass index; GLD: glucose-lowering drugs; CKD: chronic kidney disease; FPG: fasting plasma glucose.  **Model 1:** adjusted for age and sex. **Model 2**: Model 1+ further adjusted for BMI, educational level, current smoking (at first follow-up), GLD use (at baseline or first follow-up), family history of premature CVD, hypertension, hypercholesterolemia, CKD, and FPG. | | | | |
