# Supplementary material for: Weight change and risk of cardiovascular disease among adults with type 2 diabetes: more than 14 years of follow-up in the Tehran Lipid and Glucose Study
Source: Cardiovasc Diabetol. 2021 Jul 12;20:141. doi: 10.1186/s12933-021-01326-2 (PMC8276460; doi:10.1186/s12933-021-01326-2)
Supplement: Supplementary file 3 — Additional file 3: Table S3. Multivariable hazard ratios (HR) and 95% confidence intervals (CI) of association between weight change categories and incident CHD with imputed baseline missing data for covariates (number: 1104 participants): Tehran Lipid and Glucose Study, Iran, 1999–2018. [file 12933_2021_1326_MOESM3_ESM.docx]

| **Table S3. Multivariable hazard ratios (HR) and 95% confidence intervals (CI) of association between weight change categories and incident CHD with imputed baseline missing data for covariates (number: 1104 participants): Tehran Lipid and Glucose Study, Iran, 1999-2018.** | | | | |
| --- | --- | --- | --- | --- |
|  | **Model 1** | | **Model 2** | |
|  | **HR (95% CI)** | **P-value** | **HR (95% CI)** | **P-value** |
| **Weight change categories** |  |  |  |  |
| - **Lost >5%** | 1.16 (0.85-1.59) | 0.354 | 1.16 (0.84-1.59) | 0.379 |
| - **Lost 3% to 5%** | 0.82 (0.55-1.24) | 0.352 | 0.75 (0.49-1.13) | 0.169 |
| - **Stable (±3%)** | Reference |  | Reference |  |
| - **Gained 3% to 5%** | 0.72 (0.46-1.10) | 0.128 | 0.81 (0.53-1.26) | 0.352 |
| - **Gained >5%** | **0.63 (0.43-0.91)** | **0.013** | **0.62 (0.43-0.90)** | **0.012** |
| **Age, year** | **1.04 (1.03-1.05)** | **<0.001** | **1.03 (1.01-1.04)** | **<0.001** |
| **Women (Men as reference)** | **0.79 (0.62-0.99)** | **0.048** | **0.65 (0.50-0.86)** | **0.002** |
| **BMI, kg/m^2^** |  |  | 0.98 (0.96-1.01) | 0.237 |
| **Educational level, years** |  |  |  |  |
| - **>12** |  |  | Reference |  |
| - **6-12** |  |  | 0.99 (0.59-1.66) | 0.980 |
| - **<6** |  |  | 1.04 (0.62-1.75) | 0.877 |
| **Current smoker, yes** |  |  | 1.40 (0.92-2.15) | 0.119 |
| **GLD use, yes** |  |  | **1.84 (1.40-2.40)** | **<0.001** |
| **Family history of premature CVD, yes** |  |  | 1.06 (0.79-1.44) | 0.691 |
| **Hypertension, yes** |  |  | **1.73 (1.34-2.23)** | **<0.001** |
| **Hypercholesterolemia, yes** |  |  | **1.86 (1.36-2.56)** | **<0.001** |
| **CKD, yes** |  |  | 0.78 (0.59-1.03) | 0.081 |
| **FPG, mmol/L** |  |  | **1.05 (1.02-1.09)** | **0.004** |
| CHD: coronary heart disease; BMI: body mass index; GLD: glucose-lowering drugs; CVD: cardiovascular disease; CKD: chronic kidney disease; FPG: fasting plasma glucose.  **Model 1:** adjusted for age and sex. **Model 2**: Model 1+ further adjusted for BMI, educational level, current smoking (at first follow-up), GLD use (at baseline or first follow-up), family history of premature CVD, hypertension, hypercholesterolemia, CKD, and FPG. | | | | |
